# *Ki*MoSys: a web-based repository of experimental data for KInetic MOdels of biological SYStems

**DOI:** 10.1186/s12918-014-0085-3

**Published:** 2014-08-13

**Authors:** Rafael S Costa, André Veríssimo, Susana Vinga

**Affiliations:** 1 Instituto de Engenharia de Sistemas e Computadores, Investigacão e Desenvolvimento (INESC-ID), R Alves Redol 9, Lisboa, 1000-029, Portugal; 2 Center for Intelligent Systems, LAETA,IDMEC, Instituto Superior Técnico, Universidade de Lisboa, Av. Rovisco Pais 1, Lisboa, 1049-001, Portugal

**Keywords:** Dynamic modeling, Repository, Data sharing, Accessible dynamic data, Associated kinetic models

## Abstract

**Background:**

The kinetic modeling of biological systems is mainly composed of three steps that proceed iteratively: model building, simulation and analysis. In the first step, it is usually required to set initial metabolite concentrations, and to assign kinetic rate laws, along with estimating parameter values using kinetic data through optimization when these are not known. Although the rapid development of high-throughput methods has generated much omics data, experimentalists present only a summary of obtained results for publication, the experimental data files are not usually submitted to any public repository, or simply not available at all. In order to automatize as much as possible the steps of building kinetic models, there is a growing requirement in the systems biology community for easily exchanging data in combination with models, which represents the main motivation of *Ki*MoSys development.

**Description:**

*Ki*MoSys is a user-friendly platform that includes a public data repository of published experimental data, containing concentration data of metabolites and enzymes and flux data. It was designed to ensure data management, storage and sharing for a wider systems biology community. This community repository offers a web-based interface and upload facility to turn available data into publicly accessible, centralized and structured-format data files. Moreover, it compiles and integrates available kinetic models associated with the data.

*Ki*MoSys also integrates some tools to facilitate the kinetic model construction process of large-scale metabolic networks, especially when the systems biologists perform computational research.

**Conclusions:**

*Ki*MoSys is a web-based system that integrates a public data and associated model(s) repository with computational tools, providing the systems biology community with a novel application facilitating data storage and sharing, thus supporting construction of ODE-based kinetic models and collaborative research projects.

The web application implemented using Ruby on Rails framework is freely available for web access at http://kimosys.org, along with its full documentation.

## Background

Biochemical network modeling is a fundamental aspect of systems biology as a tool for performing experiments *in silico*, which has a direct application in the biotechnological and biomedical fields [1]. In particular, the fields of Metabolic Engineering and health take advantage of computational models of cell metabolism, in order to discover optimal sets of genetic manipulations for the design of mutant microbial strains that efficiently produce compounds of industrial interest and to find potential drug targets [2],[3]. Furthermore, predictive mathematical models are also important to understand complex and large amounts of *omics* data that are increasingly being collected.

One of the most common approaches for modeling metabolic pathways is the creation of mechanistic dynamic models based on ordinary differential equations (ODEs). *In vivo* kinetic modeling of metabolism requires experimental information on concentrations of metabolites, flux measurements and enzyme levels under a number of different conditions [4]. The main challenge in building such kinetic models is to choose the detailed kinetic rate expressions and their parameters [5], which have not been experimentally determined because they are difficult or even impossible to measure directly. Specific experimental standard-conditions may be required for estimating kinetic parameters [6].

While network information has been compiled in public databases [7], there are currently limited methods for measuring kinetic parameters [8]. Usually, for a large number of enzymes, the *in vivo* kinetic parameters are unknown or are only available in the literature and databases as general values obtained by *in vitro* measurements by enzymologists [9]. These parameters should be used with care by modelers, since enzymologists in general work under optimal conditions for the enzyme and do not perform the enzyme characterization under physiological conditions where a great amount of interaction is usually present [10], which severely restricts their *in silico* applicability [11],[12]. Therefore, an alternative approach to address this issue is to make use of a variety of *in vivo* data that usually includes changed intracellular metabolite concentrations and metabolic fluxes obtained from perturbations of controlled cultures [13]–[16]. These data are then used to fit the model by minimizing an objective function applying a variety of nonlinear optimization tools [17].

Due to the large amount of experimental data required to estimate these parameters, this modeling approach has been limited to key pathways of some organisms, such as *E. coli* and *S. cerevisae*. In order to overcome some of the current limitations in metabolic modeling, new approaches have been recently emerging based on the model complexity reduction [18],[19] and approximative kinetic rate formats such as linlog [20] and convenience kinetics [21]. Two recent examples have attempted to apply these approaches with integrating metabolomics measurements and flux data. Jamshidi and Palsson [22] proposed an approximate modeling approach composed of mass-action kinetics by integrating high-throughput data from several *omics* (metabolomic, fluxomic and proteomic). In [23] the authors build a dynamic model of Hepatoma cells using linlog kinetics, where the elasticity parameters are estimated from time-series metabolites data.

In order to facilitate sharing of modeling resources among researchers, several tools and standards have been constructed. These include standards for representing models (e.g., SBML [24] and CellML [25]) and for reporting experimental results (e.g., MIAME [26]). Databases freely available are also important resources for information sharing, which can be used to build a model. Examples are the pathway information, such as KEGG [27] and BioCyc [28], and enzyme kinetics available in e.g., SABIO-RK [29] and BRENDA [9]. Furthermore, established kinetic metabolic models can be found in publicly available repositories (e.g. JWS online [30] and Biomodels [31]) and several tools (e.g., Systems Biology Toolbox [32], COPASI [33] and CellDesigner [34]) have been developed to simulate and analyze these models. Recently, many efforts have converged to encourage sharing data, workflows, and analytical codes, to promote overall reproducibility [35]–[37]. Despite all resources and tools, there is a growing requirement for disseminate of experimental data and related protocols [38], kinetic models, as well as applications to support the construction of large-scale kinetic models for the wider systems biology community. In this respect, *Ki*MoSys system has several distinguishing features: i) it provides a public repository of annotated and structured-data required for kinetic modeling, ii) it contains metadata information to establish essential properties about the data such as the corresponding experimental and environmental conditions iii) it allows to associate kinetic models with the data and supports a model intermediate files history system and iv) it can support researchers on the kinetic building process of metabolic networks.

Public access to datasets and their standardization are requirements for the reproduction of research finding [39], for complementing experiments between different labs and successful kinetic pathway modeling [40]. Moreover, the description of the data protocols properties may encourage further research collaborations between different laboratories. However, experimentalists usually use unstandardized file-based management systems locally on their personal computers to manage their results - a strategy that is ill-suited for the requirements of data sharing in systems biology research. Further disadvantages are also the unavailability of a system to associate kinetic models with experimental data and the lack of the kinetic model development history. Hence, an intuitive repository which offers researchers the access to steady-state and dynamic data would be of great help, as widely recognized by the systems biology community of this field [41],[42].

In this work, we present an open source repository of experimental data (for stationary and dynamic conditions) and related information from the literature, which is oriented towards building kinetic modeling and supporting modellers and experimentalists, in the very time consuming process of collection data and corresponding information from different publications. Moreover, it integrates tools to support and facilitate the first steps of kinetic models construction process of large-scale metabolic networks for non-experts users.

## Construction and content

### Data collection

*Ki*MoSys is a web-based platform developed in Ruby on Rails framework (http://rubyonrails.org/). To facilitate the access and use of published experimental data required for modeling biochemical networks, we have collected publicly available metabolite concentrations, enzyme levels and flux data, and made them accessible on our *Ki*MoSys repository. These experimental data were provided by the authors upon our request or acquired through internet search (from the supplementary material when available) and are referenced by the origin of the data. Before being added to the repository, the data were reformatted according to an Excel (which is the most utilized and familiar data format in Systems Biology for lab researchers to collect and share their various types of data) template file to make the data available and widely usable. To overcome different nomenclature conventions – for instance, for the match between the data produced by different researchers and data needed in computational models - annotation of the biochemical elements with specific database identifiers was incorporated and linked in the structured Excel file. The biological ontologies used are ChEBI [43], KEGG [27], UniProt ID’s [44] and NCBI organism taxonomy [45]. Based on these annotations links to external databases enables the user to obtain further details. On the other hand, the database also allows the submission of different kinetic models for the same data. The associated models were taken from the BioModels database [31], JWS online [30], manually retrieved from additional files of the original paper or provided by the authors. Detailed information extracted and collected from the original papers is also provided with each dataset and associated model. All this information was stored in a SQLite v3 (http://www.sqlite.org) database. *Ki*MoSys repository will be routinely updated and expanded as new dataset and models become available. At the present time, 36 experimental datasets from 31 different publications and for 13 different organisms (including *Escherichia coli*, *Saccharomyces cerevisiae* and *Lactococcus lactis*) as well as 12 associated kinetic models have been submitted to the repository (see Additional file 1). In the future it is expected that these numbers will increase.

### Implementation and system architecture

The application is designed as a web-based client-server application model. It was developed using Ruby on Rails full stack web application framework that uses a Model-View-Controller architecture. In particular, it uses an implementation of Ruby on Rails (http://rubyonrails.org/) that allows for a transparent integration with Java tools and in the future the possibility to extend to libraries in other languages. The architecture can be separated in three layers, (i) interface; (ii) application logic; and (iii) data storage.

*Ki*MoSys is a web-based application, and it uses web’s modern technologies and techniques, such as html, css and javascript to deliver an user-friendly experience. Accordingly, users are able to rapidly take advantage of the full functionality of the application by using established design patterns.

*Ki*MoSys application logic is implemented in ruby programming language and uses Java libraries to expose core functions to the community. These libraries were previously developed and this functionality can be easily extended taking advantage of other existing or new libraries. The source code for *Ki*MoSys is available at the documentation section (http://kimosys.org/documentation).

The application’s storage implements a SQLite database to leverage its portability and is an integral part of the application as it does not require additional processes to be running.

## Utility and discussion

### *Ki*MoSys overview

*Ki*MoSys was designed to provide published experimental data and links to corresponding associated kinetic models, and tools to facilitate the development of large-scale kinetic models. Specifically, the web-platform can be divided into two main parts: i) access to uniformily annotated data files (including metadata to make data comparable) with a focus on supporting kinetic modeling, and ii) user-friendly tools to facilitate the building of large-scale kinetic models. The combination of these features into one freely available web-tool represents its main utility and novelty.

### Contents and main features

**Figure 1:**
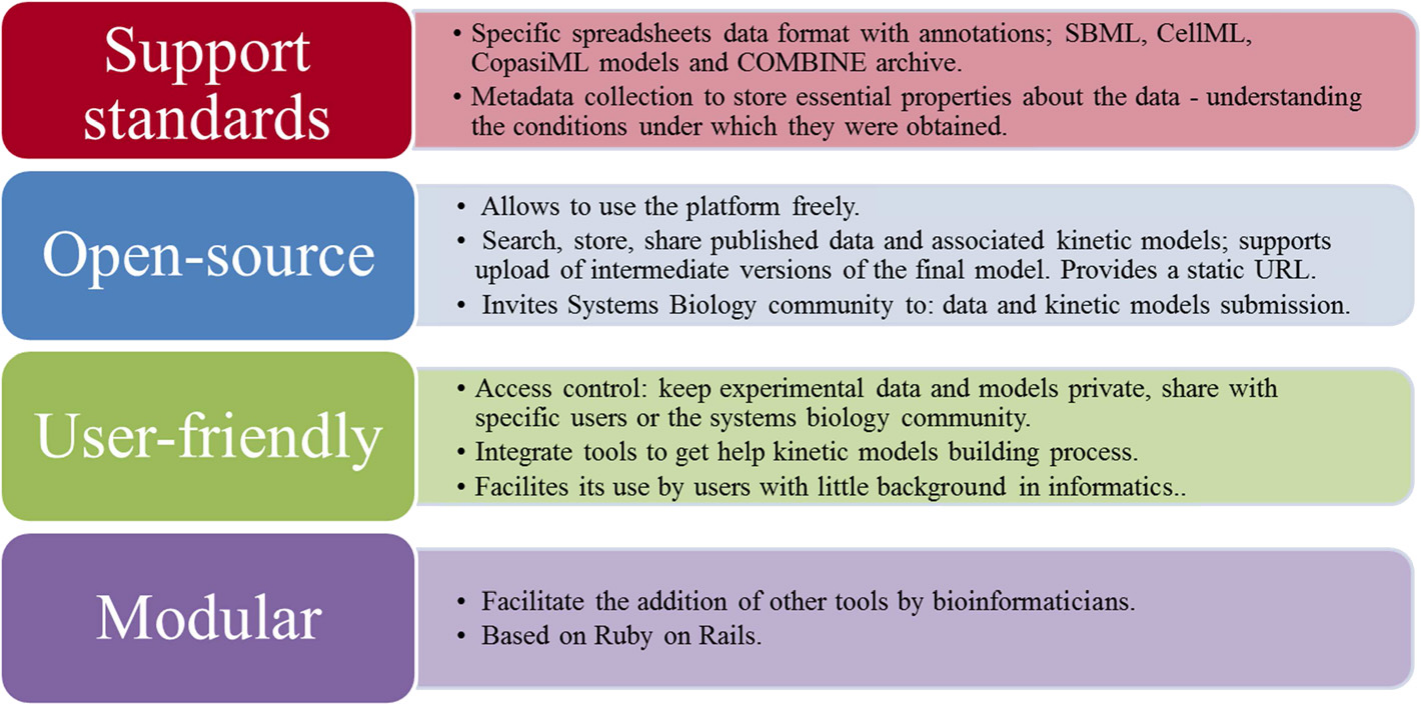
**Main features of** ***Ki*** **MoSys.**

Major features of the platform are shown in Figure 1 and are described in more detail in the following sections.

Users can access the *Ki*MoSys repository to search published data and associated kinetic models (i.e., models that used a particular dataset for model construction and/or validation), and organize and share their own published data. At the moment, the repository is focused on the most commonly used data types (metabolites data, enzyme concentrations and flux measurements) in kinetic modeling. Overall, *Ki*MoSys repository is a flexible platform allowing users to access experimental data that is usually not accessible (e.g. as supplementary material) in the original paper. Regularly, we will invite authors (system biologists and modelers) to submit data and associated model files directly to the repository, and consequently extending the content of the repository. In our platform it is possible to associate different kinetic models with the same data. We included this possibility, because it is important to point out the experimental data on which the kinetic model building/validation is based. This feature is fully compliant to the COMBINE community (http://co.mbine.org/) efforts that provide the COMBINE archive (a single file containing various documents for the description a model and associated data). Data and models can be submitted from older published articles by any community member whether or not being an author on the paper. In the future, we want to provide a special service in cooperation with several journals that ask the authors if they would like to deposit their experimental data and associated kinetic models in the *Ki*MoSys repository at the same time of submitting a publication.

To facilitate the kinetic model building of large metabolic networks, we also provide some useful tools for this modeling step. Due to its modular architecture based on Ruby, *Ki*MoSys is very flexible and facilitate the addition of other tools/algorithms by systems biologists.

### Structure and user accessibility of the repository

**Figure 2:**
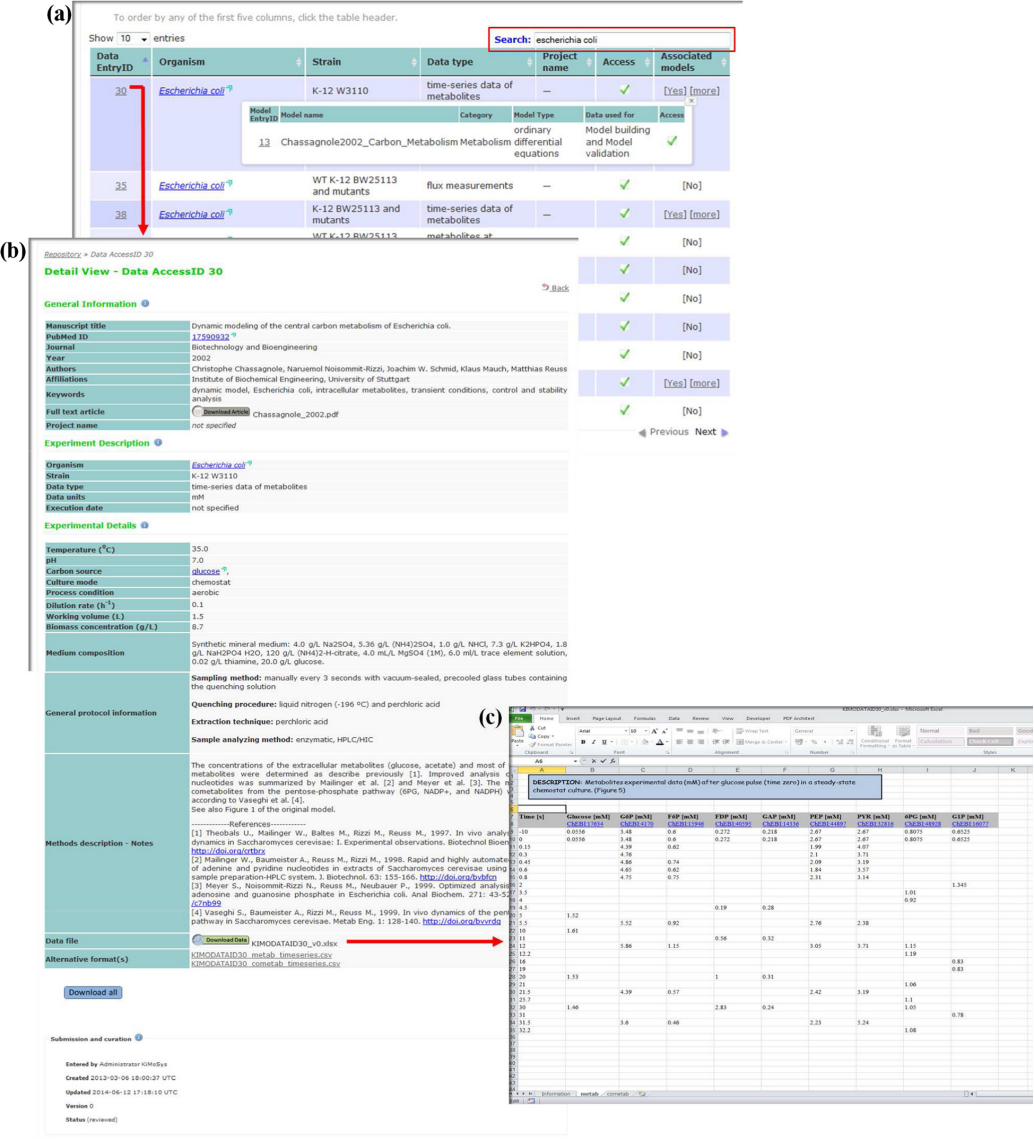
**Screenshots of*Ki*MoSys respository. (a)** Results of “*Escherichia coli*” search in the *Ki*MoSys database; **(b)** example of a detailed view window from *Escherichia coli* (*Data EntryID 30*) and associated model(s) table, and **(c)** corresponding worksheet of the structured Excel data file (including names, units and ChEBI ID’s for annotation) obtained from the download.

To compile the stored data in an accessible and logical manner, and to organise the data we have created a hierarchical structure. In addition, we adapted the main principles recommended for the design and maintenance of biological databases [46]. This was accomplished by using a top-level structure with five main index contents (see Figure 2a). Examples of the main types of simple queries supported in this release are the following: (i) *data EntryID* – a unique number assigned automatically after successful submission of an data file (ii) *organism* - the name of the organism used to generate the dataset (iii) *data type* - the experimental data type available, and (iv) PubMed ID – ID reference from PubMed where the data or model are described.

The detail view structure contains the metadata about the data (Figure 2b). The first section contains basic information about the manuscript where the data are published (e.g. *title*, *authors*, *affiliations*, *keywords*, etc.) and is linked with the original manuscript (source of information) in order to facilitate the research process. The second part contains the experimental description: organism, strain, data type and units, and execution date. To account for experimental dependencies, we also include experimental details, such as the environmental conditions under which the cells were grown or which method was used to make the measurement of the data. This minimum information level of detail is a key point to enable reproducibility and comparisons of the experiment reported in the paper. The section holds also the *process condition*, *medium composition*, *protocol description* and a direct link to the Excel data file for download (Figure 2c). In addition, in the interest of interoperability in Systems Biology, a recommended data table style structure SBtab (http://www.sbtab.net) is adopted and provided as part of the normal Excel data file. These tables can then be converted and inserted in the commonly SBML (Systems Biology Markup Language) exchange format, allowing its import into different modeling tools supporting SBML. The *Ki*MoSys repository is also flexible enough to accommodate interesting requirements in the future without a major overhaul. Associated models per *Data EntryID*, have similar main fields such as *Data EntryID*, model name, category and model type. The possibility of associating the model with the data allows the user to have the knowledge about the data used to calibrate and validate the model(s), but also information on the data provenance.

The permission rules are applied to the data and also to the coupled kinetic models. Note that the data submitter can be different from the model submitter. These can remain public and private for all users or can be shared with individual users (e.g. project memberships, journal reviewers and editors during peer review). Unregistered users are allowed to browse, view the public information and download the public experimental data and the corresponding associated kinetic models. Registered users (upon authorized login) are able to browse, upload, share and download the public available data and model files. Moreover, submitters per *EntryID* entity are able to invite scientific project collaborators, keep the data file private and update assigned data to the repository. All of the submitted files per *EntryID* are private by default. In a paper review process and after a manuscript is accepted for publication, the private files can be changed to public by the submitter. Invited users have also access to their private data and model files. This functionality promotes collaborative projects, in which experimentalists collect the data, may publish them for colleagues working in different labs, and then the overall results and data will be published and linked.

### Repository contributions

**Figure 3:**
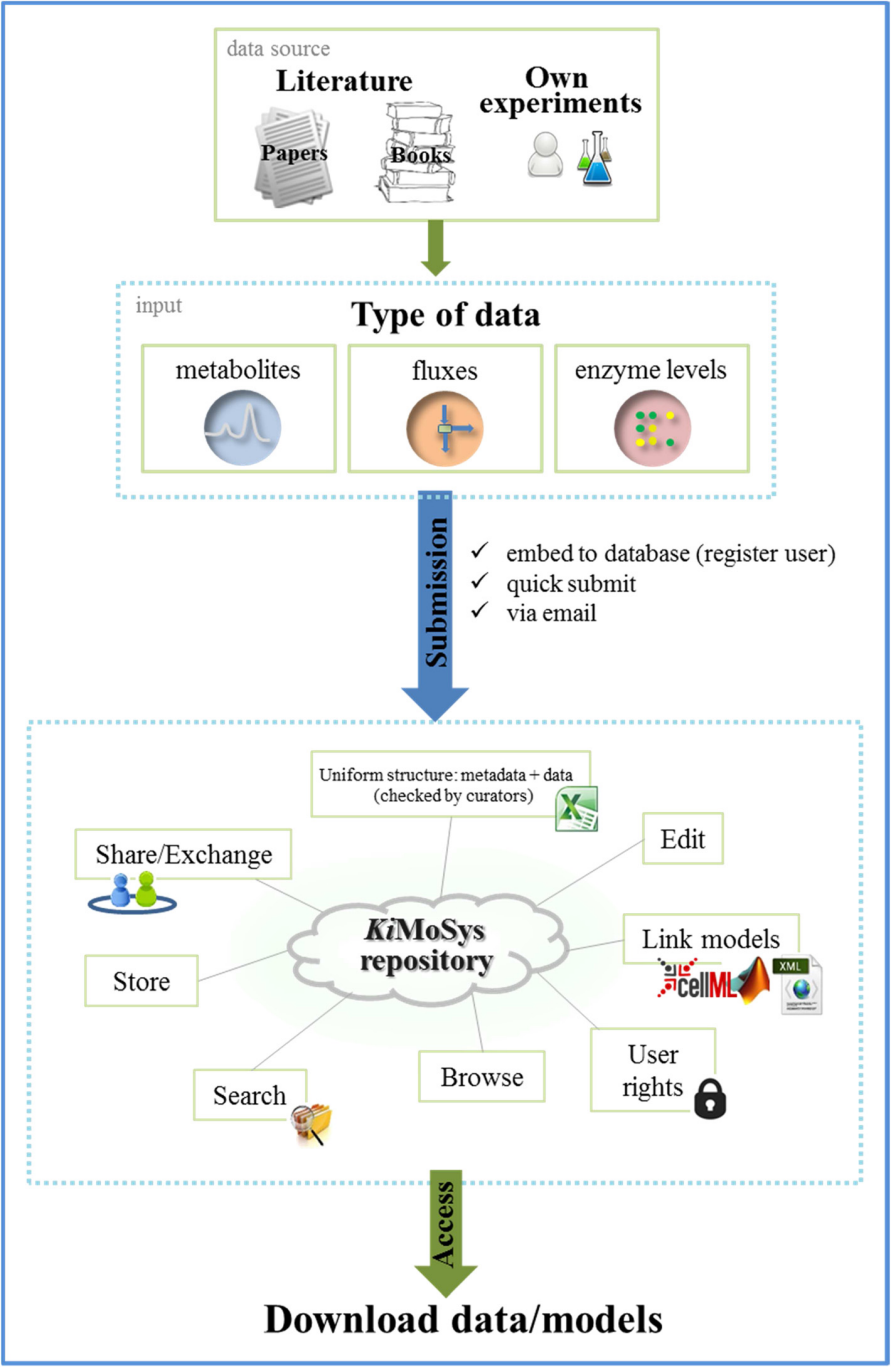
**Overview of the** ***repository*** **workflow schema.**

The repository is equipped with an online submission interface (electronic data-submission and quick submit) and a paper form submission to insert data. This allows users to describe the data in terms of conditions and protocol information (to understand the conditions under which they were obtained), structure it in one consistent and popular format in Systems Biology (Excel spreadsheets), and share their experimental data files with the community (Figure 3). The submission form presents metadata information by general information, experiment description and experimental details. The workflow not only includes the data and models collected from the literature but also manually curated work to complete and validate the information for the data storage.

An online submission form to associate models with existing data is also available, that enables researchers to specify the details of the model content (i.e. model type, number of reactions, species, parameters, etc.). In addition, it supports the upload of several intermediate and individual files of the final model by providing a simple history system that offers access to all files and a possibility to comment each change (development history). This represents an important feature, because the kinetic model building process consists in an iterative task producing several intermediate versions and plays an important role in understanding the justification for certain parts of a model. Administrators and submitters are notified via email when data and/or models are submitted to the repository and the project collaborators are notified when they are invited to a specific data/model *AccessID*. One additional advantage of *Ki*MoSys repository is the amount of metadata it contains, which is a key feature to put the data in biological/technical context.

### Computational tools

**Figure 4:**
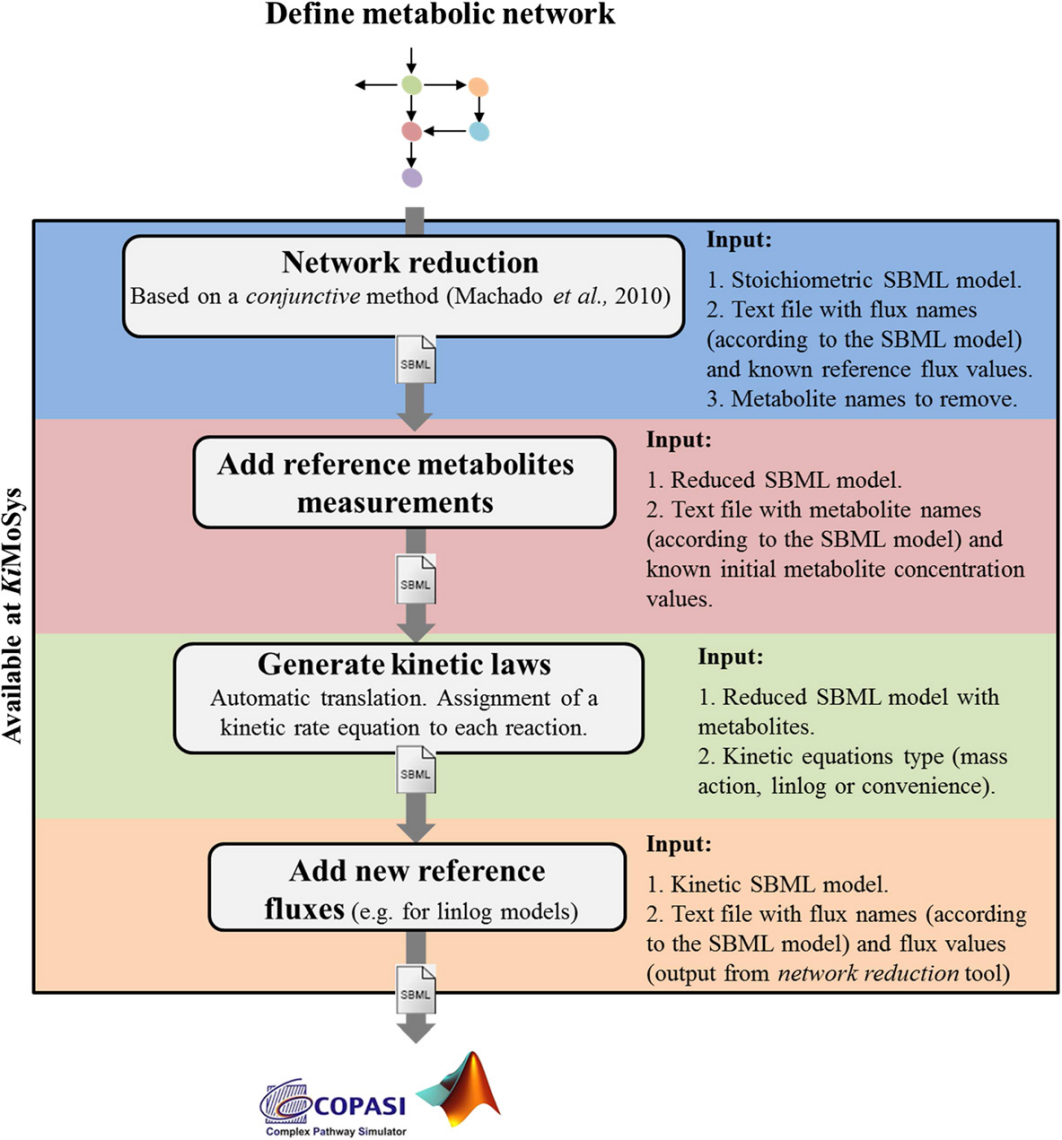
**The*Ki*MoSys*tools*workflow.** The workflow starts with the definition of a metabolic network (SBML model). Then this can be loaded in the *network reduction* tool (step 1). The output is a reduced model file. Subsequently, the user can add all the initial metabolites values that are contained in the model (step 2). The *Translate kinetic equation*s tool (step 3), allows to be assigned approximated kinetics (convenience, lin-log and mass-action) to the corresponding reactions in the SBML model. *Add fluxes* tool (step 4) use as input the new fluxes file from step 2 and set all the reference fluxes for each lin-log rate law in the reduced SBML model.

*Ki*MoSys also provides a number of applications (*tools* section) to support and speed up the first kinetic modeling steps for large-scale metabolic networks, including model network reduction and semi-automated kinetic rate equations generation. These tools are depicted in Figure 4 as a workflow.

In order to demonstrate the utility of the *Ki*MoSys *tools*, we have chosen the *E. coli* core network described in [47] as a case study. In this section we present the basic details of the workflow:

1. Select a reconstructed metabolic network (stoichiometric model in SBML format) you wish to study. Here, the SBML file with the core metabolic network of *E. coli* was taken from http://systemsbiology.ucsd.edu/InSilicoOrganisms/Ecoli/EcoliSBML. The metabolic network has 63 metabolites and 77 reactions.

2. An important requisite for kinetic modeling is the model complexity reduction [48],[49]. The key motivation for model reduction is the need for simple models that nevertheless capture the important features of complex dynamic systems. We provided a model reduction algorithm in *Ki*MoSys whose major features is the ability to reduce the model *a priori*, i.e. before the parameter estimation task. This is based on the steady-state time scale analysis and is described in detail in [50]. The main limitation in this model reduction approach is the selection of the metabolites, since selecting a limited set of metabolites from a ranked list is usually arbitrary. The *model reduction* tool requires three input files. The first is the stoichiometric SBML model, here exemplified for the core network of *E. coli*. The second is a text file containing, both flux names and the corresponding known flux distribution values. Here, we use Flux Balance Analysis (FBA) to estimate the reference flux distribution using the COBRA toolbox [51]. The linear programming problem was solved for maximizing of biomass production with the glucose consumption rate (GLCpts) and maintenance energy requirement rate (ATPM) as constraints fixed in 0.2004 mM s^−1^ (experimental value taken from [52]) and 1.193 mM s^−1^ (experimental data taken from [53]), respectively. The reaction directionality for the negative fluxes was reversed, so that the reference fluxes are all positive (see example file 2 available on the tools tab). The third input is the metabolite names to be removed from the network. The output consists of one compressed zip archive containing two files: the first file contains the reduced SBML model and the second consists of results from the new flux distribution of the reduced network.

3. To construct a kinetic model, initial metabolite concentration values must be provided for all metabolites in the network. For this case study, the reference initial concentration values were taken from the literature for *E. coli* at D = 0.1 h^−1^[14],[52],[54] and available on the repository. The unknown metabolites were set to the median concentration value of *E. coli* at D = 0.1 h^−1^ ~ 0.0221 mM [52]. The values used for the initial metabolite concentrations are given in (see example file 2 available on the tools tab). The use of *Add metabolites* tool requires two input files: (i) the SBML model, for this case we use the reduced SBML file (output from the *model reduction* tool) and (ii) the text file with the initial known metabolite concentration and the corresponding name. The output contains the SBML model with the initial metabolite concentration values.

4. To turn a stoichiometric model into a kinetic model, rate laws have to be assigned for each reaction. Therefore, a tool that semi-automatically generates and assigns all the rate laws to the model reactions, at the same time in the approximate linlog kinetic format or in other types of approximate kinetic rate laws (mass action and convenience kinetics) is implemented. The values of kinetic parameters and compartment size were set to 1.0. For the linlog kinetics the parameter values were initialized with the negative of the corresponding stoichiometric coefficients. To generate kinetic laws the tool *translate kinetic equations* requires only the reduced SBML model with metabolites and the approximate rate equation type (for the case study we select linlog kinetics).

5. Similar to *add metabolites* tool, the *set fluxes* requires two input files: (i) the reduced kinetic model obtained from step 3 and (ii) a text file with known flux distribution values and the corresponding names (output file from the *model reduction* tool).

After defining all the initial metabolite concentrations, kinetic rate equations and fluxes, the exported *draft* kinetic model (SBML file) is ready for further parameterization (for example, parameter fitting using experimental data available on the *repository*) and analysis in external tools such as the COPASI [33] and the SBToolbox for Matlab [32]. Finally, all the model versions can be submitted to the *Ki*MoSys *repository*.

This case study shows how we can use these tools to help for the semi-automated kinetic model generation of large scale metabolic networks. Although the workflow is focusing on this case study it can be applied to metabolic reconstructions from any organisms. Moreover, it is also possible to upload directly the data and model files stored in the repository. The developed software pipeline composed of several steps can be run sequentially or individually.

### Challenges and future directions

The future work of *Ki*MoSys is to provide an integrated platform that enables users to access experimental data and supports the overall kinetic modeling tasks, so that tools that are used at different stages of the computational workflow can be easily used together. New features of *Ki*MoSys will potentially be able to automate as much as possible the kinetic model editing and building process, as well as to integrate with other tools (e.g. CopasiWS Web service [55]), to perform the simulation and analysis steps. In the next *Ki*MoSys release an extension to export the data directly into the alternative RDF (http://www.w3.org/RDF/) and SBML file formats will be included. Moreover, we aim to add support of MIRIAM [56] compliant annotations for the generated SBML models in the *Ki*MoSys tools. Apart from this, *Ki*MoSys repository will be routinely expanded as new datasets become available.

## Conclusions

Computational modeling to create kinetic models of biochemical networks requires experimental information under different conditions. For this purpose we have built *Ki*MoSys a web-based repository oriented to kinetic modeling that provides a public data repository of published data in a structured format including annotations to external databases, and associated kinetic models, to ensure high data access and exchange between modelers and experimentalists. Furthermore, *Ki*MoSys integrates tools to support and facilitate the kinetic model construction process of large-scale metabolic networks.

## Availability and requirements

**Project name**: *Ki*MoSys

**Project home page**: http://kimosys.org

**Operating system**: platform independent

**Programming language**: Ruby and Java

**License**: GNU GPL v2

**Any restrictions to use by non-academics**: only those imposed by the license.

## Competing interests

The authors declare that they have no competing interests.

## Authors’ contributions

RC and AV implemented the application, RC collected and added the experimental data and kinetic models to *Ki*MoSys repository, RC designed the application, and drafted the manuscript. All authors read and approved the final manuscript.

## Additional file

## Supplementary Material

Additional file 1: **Data and associated models available in *Ki*** **MoSys repository.** Click here for file
