# Supplementary material for: KiMoSys: a web-based repository of experimental data for KInetic MOdels of biological SYStems
Source: BMC Syst Biol. 2014 Aug 13;8:85. doi: 10.1186/s12918-014-0085-3 (PMC4236735; doi:10.1186/s12918-014-0085-3)
Supplement: Additional file 1: — Data and associated models available in Ki MoSys repository. [file s12918-014-0085-3-S1.pdf]

Table 1. Data and associated models available in *Ki*MoSys repository.

Data EntryID	Organism	Type of data	Original Publication Title	Reference	Reference of associated model(s)
30	*Escherichia coli*	time-series data of metabolites	Dynamic modeling of the central carbon metabolism of *Escherichia coli*.	[1]	[1]
35	*Escherichia coli*	flux measurements	Multiple high-throughput analyses monitor the response of *E. coli* perturbations.	[2]	-
37	*Lactococcus lactis*	time-series data of metabolites	Is the glycolytic flux in *Lactococcus lactis* primarily controlled by the redox charge? Kinetics of NAD(+) and NADH pools determined *in vivo* by ^{13}C NMR.	[3]	[4,5]
38	*Escherichia coli*	time-series data of metabolites	Modeling and simulation of the main metabolism in *Escherichia coli* and its several single-gene knockout mutants with experimental verification	[6]	[6]
41	*Escherichia coli*	metabolites at steady-state	Multiple high-throughput analyses monitor the response of *E. coli* perturbations.	[2]	-
42	*Clostridium acetobutylicum*	time-series data of metabolites	A systems biology approach to investigate the effect of pH-induced gene regulation on solvent production by *Clostridium acetobutylicum* in continuous culture.	[7]	[7]
44	*Escherichia coli*	enzyme/protein concentrations	Multiple high-throughput analyses monitor the response of *E. coli* perturbations	[2]	-
51	*Escherichia coli*	time-series data of metabolites	Dynamic simulation of an *in vitro* multi-enzyme system.	[8]	-
52	*Escherichia coli*	metabolites at steady-state	Metabolic profiling of *Escherichia coli* cultivation: evaluation of extraction and metabolite analysis procedures.	[9]	-
54	*Escherichia coli*	metabolites at steady-state	Quantification of intracellular metabolites in *Escherichia coli* K12 using liquid chromatographic-electrospray ionization tandem mass spectrometric technique.	[10]	-
55	*Pichia pastoris*	metabolites at steady-state	Glucose-methanol co-utilization in *Pichia pastoris* studied by metabolomics and instationary ^{13}C flux analysis.	[11]	-
57	*Pichia pastoris*	flux measurements	Glucose-methanol co-utilization in *Pichia pastoris* studied by metabolomics and instationary ^{13}C flux analysis.	[11]	-
58	*Homo sapiens*	metabolites at steady-state	Dynamics and control of the central carbon metabolism in Hepatoma cells.	[12]	-

(continued from previous page)

Data EntryID	Organism	Type of data	Publication Title	Reference	Reference of associated model(s)
59	*Rattus*	time-series data of metabolites	The selective control of glycolysis, gluconeogenesis and glycogenesis by temporal insulin patterns.	[13]	-
61 and 62	*Saccharomyces cerevisiae*	time-series data of metabolites	Temporal system-level organization of the switch from glycolytic to gluconeogenic operation in yeast.	[14]	-
63	*Escherichia coli*	time-series data of metabolites	Metabolomics-driven quantitative analysis of ammonia assimilation in *E. coli*.	[15]	[15]
64	*Escherichia coli*	flux measurements	Impact of global transcriptional regulation by arcA, arcB, cra, crp, cya, fnr, and mlc on glucose catabolism in *Escherichia coli*.	[16]	-
65	*Escherichia coli*	flux measurements	Nonlinear dependency of intracellular fluxes on growth rate in miniaturized continuous cultures of *Escherichia coli*.	[17]	-
66	*Shewanella oneidensis*	flux measurements	Invariability of central metabolic flux distributions in *Shewanella oneidensis* MR-1 under environmental or genetic perturbation	[18]	-
67 and 68	*Escherichia coli*	flux measurements	An integrated computational and experimental study for overproducing fatty acids in *Escherichia coli*.	[19]	-
69 and 70	*Saccharomyces cerevisiae*	time-series data of metabolites	Characterization of glucose transport mutants of *Saccharomyces cerevisiae* during a nutritional upshift reveals a correlation between metabolite levels and glycolytic flux.	[20]	[20]
71	*Penicillium chrysogenum*	time-series data of metabolites	Generating short-term kinetic responses of primary metabolism of *Penicillium chrysogenum* through glucose perturbation in the bioscope mini reactor.	[21]	-
72	*Aspergillus niger*	flux measurements	Integration of in vivo and in silico metabolic fluxes for improvement of recombinant protein production.	[22]	-
73	Aspergillus niger	flux measurements	Overexpression of isocitrate lyase-glyoxylate bypass influence on metabolism in *Aspergillus niger*.	[23]	-
74	*Escherichia coli*	metabolites at steady-state	Catching prompt metabolite dynamics in *Escherichia coli* with the BioScope at oxygen rich conditions.	[24]	-
75	*Escherichia coli*	time-series data of metabolites	Changes in substrate availability in *Escherichia coli* lead to rapid metabolite, flux and growth rate response.	[25]	-
76	*Streptomyces coelicolor*	time-series data of metabolites	Intracellular metabolite pool changes in response to nutrient depletion induced metabolic switching in *Streptomyces coelicolor*.	[26]	-

(continued from previous page)

Data EntryID	Organism	Type of data	Publication Title	Reference	Reference of associated model(s)
77	*Shewanella oneidensis*	time-series data of metabolites	Integrating flux balance analysis into kinetic models to decipher the dynamic metabolism of *Shewanella oneidensis.*	[27]	[27]
78	*Escherichia coli*	time-series data of metabolites	Metabolic flux analysis in a nonstationary system: fed-batch fermentations of a high yielding strain of *E. coli* producing 1,3-propanediol.	[28]	-
79	*Escherichia coli*	metabolites at steady-state	Metabolic regulation analysis of wild-type and arcA mutant *Escherichia coli* under nitrate conditions using different levels of omics data.	[29]	-
80	*Escherichia coli*	flux measurements	Metabolic regulation analysis of wild-type and arcA mutant *Escherichia coli* under nitrate conditions using different levels of omics data.	[29]	-
81	*Mouse*	enzyme/protein concentrations	Division of labor by dual feedback regulators controls JAK2/STAT5 signaling over broad ligand range.	[30]	[30]
82	*Homo sapiens*	enzyme/protein concentrations	Network quantification of EGFR signaling unveils potential for targeted combination therapy.	[31]	-

Data EntryID: internal accession identifier.
Data EntryID without associated models is indicated by “-”.

Reference List

1. Chassagnole C, Noisommit-Rizzi N, Schmid JW, Mauch K, Reuss M: **Dynamic modeling of the central carbon metabolism of *Escherichia coli*.** *Biotechnology and Bioengineering* 2002, **79:** 53-73.

2. Ishii N, et.al.: **Multiple high-throughput analyses monitor the response of *E. coli* to perturbations.** *Science* 2007, **316:** 593-597.

3. Neves AR, Ventura R, Mansour N, Shearman C, Gasson MJ, Maycock C *et al*.: **Is the glycolytic flux in Lactococcus lactis primarily controlled by the redox charge? Kinetics of NAD(+) and NADH pools determined in vivo by C-13 NMR.** *Journal of Biological Chemistry* 2002, **277:** 28088-28098.

4. Levering J, Musters MWJM, Bekker M, Bellomo D, Fiedler T, de Vos WM *et al.*: **Role of phoshate in the central metabolism of two lactic acid bacteria - a comparative systems biology approach.** *FEBS J* 2012, **279:** 1274-1290.

5. Vinga S, Neves AR, Santos H, Brandt BW, Kooijman SALM: **Subcellular metabolic organization in the context of dynamic energy budget and biochemical systems theories.** *Philosophical Transactions of the Royal Society B-Biological Sciences* 2010, **365:** 3429-3442.

6. Kadir TAA, Mannan AA, Kierzek AM, McFadden J, Shimizu K: **Modeling and simulation of the main metabolism in *Escherichia coli* and its several single-gene knockout mutants with experimental verification.** *Microbial Cell Factories* 2010, **9:** 88.

7. Haus S, Jabbari S, Millat T, Janssen H, Fischer RJ, Bahl H *et al.*: **A systems biology approach to investigate the effect of pH-induced gene regulation on solvent production by Clostridium acetobutylicum in continuous culture.** *Bmc Systems Biology* 2011, **5**.

8. Ishii N, Suga Y, Hagiya A, Watanabe H, Mori H, Yoshino M *et al.*: **Dynamic simulation of an *in vitro* multi-enzyme system.** *FEBS* 2007, **581:** 413-420.

9. Hiller J, Franco-Lara E, Weuster-Botz D: **Metabolic profiling of Escherichia coli cultivations: evaluation of extraction and metabolite analysis procedures.** *Biotechnology Letters* 2007, **29:** 1169-1178.

10. Buchholz A, Takors R, Wandrey C: **Quantification of intracellular metabolites in *E. coli* K12 using liquid chromatographic-electrospray ionization tandem mass spectrometric techniques.** *Analytical Biochemistry* 2001, **295:** 129-137.

11. Jorda J, Suarez C, Carnicer M, ten Pierick A, Heijnen JJ, van Gulik W *et al.*: **Glucose-methanol co-utilization in Pichia pastoris studied by metabolomics and instationary C-13 flux analysis.** *Bmc Systems Biology* 2013, **7**.

12. Maier K, Hofmann U, Reuss M, Mauch K: **Dynamics and control of the central carbon metabolism in hepatoma cells.** *BMC Systmes Biology* 2010, **4:** 54-82.

13. Noguchi R, Kubota H, Yugi K, Toyoshima Y, Komori Y, Soga T *et al.*: **The selective control of glycolysis, gluconeogenesis and glycogenesis by temporal insulin patterns.** *Molecular Systems Biology* 2013, **9**.

14. Zampar GG, Kummel A, Ewald J, Jol S, Niebel B, Picotti P *et al.*: **Temporal system-level organization of the switch from glycolytic to gluconeogenic operation in yeast.** *Molecular Systems Biology* 2013, **9**.

15. Yuan J, et al.: **Metabolomics-driven quantitative analysis of ammonia assimilation in *E. coli*.** *Molecular Systems Biology* 2009, **5**.

16. Perrenoud A, Sauer U: **Impact of global transcriptional regulation by ArcA, ArcB, Cra, Crp, Cya, Fnr, and Mlc on glucose catabolism in *Escherichia coli*.** *Journal of Bacteriology* 2005, **187:** 3171-3179.

17. Nanchen A, Schicker A, Sauer U: **Nonlinear dependency of intracellular fluxes on growth rate in miniaturized continuous cultures of Escherichia coli.** *Applied and Environmental Microbiology* 2006, **72:** 1164-1172.

18. Tang YJ, Martin HG, Deutschbauer A, Huang R, Llora X, Arkin A *et al*.: **Invariability of centrl metabolic flux distribution in Shewanella oneidensis MR-1 under environmental or genetic perturbations.** *Biotechnol Prog* 2009, **25:** 1254-1259.

19. Ranganathan S, Tee TW, Chowdhury A, Zomorrodi AR, Yoon JM, Fu YF *et al*.: **An integrated computational and experimental study for overproducing fatty acids in Escherichia coli.** *Metabolic Engineering* 2012, **14:** 687-704.

20. Bosch D, Johansson M, Ferndahl C, Franzen CJ, Larsson C, Gustafsson L: **Characterization of glucose transport mutants of Saccharomyces cerevisiae during a nutritional upshift reveals a correlation between metabolite levels and glycolytic flux.** *Fems Yeast Research* 2008, **8:** 10-25.

21. Nasution U, van Gulik WM, Proell A, van Winden WA, Heijnen JJ: **Generating short-term kinetic responses of primary metabolism of Penicillium chrysogenum through glucose perturbation in the bioscope mini reactor.** *Metabolic Engineering* 2006, **8:** 395-405.

22. Driouch H, Melzer G, Wittmann C: **Integration of in vivo and in silico metabolic fluxes for improvement of recombinant protein production.** *Metabolic Engineering* 2012, **14:** 47-58.

23. Meijer S, Otero J, Olivares R, Andersen MR, Olsson L, Nielsen J: **Overexpression of isocitrate lyase-glyoxylate bypass influence on metabolism in Aspergillus niger.** *Metabolic Engineering* 2009, **11:** 107-116.

24. De Mey M, Taymaz-Nikerel H, Baart G, Waegeman H, Maertens J, Heijnen JJ *et al*.: **Catching prompt metabolite dynamics in Escherichia coli with the BioScope at oxygen rich conditions.** *Metabolic Engineering* 2010, **12:** 477-487.

25. Taymaz-Nikerel H, De Mey M, Baart G, Maertens J, Heijnen JJ, van Gulik W: **Changes in substrate availability in Escherichia coli lead to rapid metabolite, flux and growth rate responses.** *Metabolic Engineering* 2013, **16:** 115-129.

26. Wentzel A, Sletta H, Consortium S, Ellingsen TE, Bruheim P: **Intracellular metabolite pool changes in response to nutrient depletion induced metabolic switching in *Streptomyces coelicolor*.** *Metabolites* 2012, **2:** 178-194.

27. Feng X, Chen Y, Tang YJ: **Integrating flux balance analysis into kinetic models to decipher the dynamic metabolism of Shewanella oneidensis MR-1.** *PLOS Comput Biol* 2012, **8:** e1002376.

28. Antoniewicz MR, Kraynie DF, Laffend LA, Gonzalez-Lergier J, Kelleher JK, Stephanopoulos G: **Metabolic flux analysis in a nonstationary system: Fed-batch fermentation of a high yielding strain of E. coli producing 1,3-propanediol.** *Metabolic Engineering* 2007, **9:** 277-292.

29. Toya Y, Nakahigashi K, Tomita M, Shimizu K: **Metabolic regulation analysis of wild-type and arcA mutant Escherichia coli under nitrate conditions using different levels of omics data.** *Molecular Biosystems* 2012, **8:** 2593-2604.

30. Bachmann J, Raue A, Schilling M, Bohm ME, Kreutz C, Kaschek D *et al*.: **Division of labor by dual feedback regulators controls JAK2/STAT5 signaling over broad ligand range.** *Molecular Systems Biology* 2011, **7**.

31. Klinger B, Sieber A, Fritsche-Guenther R, Witzel F, Berry L, Schumacher D *et al*.: **Network quantification of EGFR signaling unveils potential for targeted combination therapy.** *Molecular Systems Biology* 2013, **9**.
